# GENetic characteristics and REsponse to lipid-lowering therapy in familial hypercholesterolemia: GENRE-FH study

**DOI:** 10.1038/s41598-020-75901-0

**Published:** 2020-11-09

**Authors:** Hyoeun Kim, Chan Joo Lee, Hayeon Pak, Doo-Il Kim, Moo-Yong Rhee, Byoung Kwon Lee, Youngkeun Ahn, Byung-Ryul Cho, Jeong-Taek Woo, Seung-Ho Hur, Jin-Ok Jeong, Ji Hyun Lee, Sang-Hak Lee

**Affiliations:** 1 Department of Health Promotion, Yonsei University College of Medicine, Seoul, South Korea; 2 Division of Cardiology, Department of Internal Medicine, Severance Hospital, Yonsei University College of Medicine, 134 Shinchon-dong, Seodaemun-gu, Seoul, 120-752 South Korea; 3 Department of Life and Nanopharmaceutical Sciences, Graduate School, Kyung Hee University, Seoul, South Korea; 4 Cardiology Division, Department of Internal Medicine, Haeundae Paik Hospital, Inje University College of Medicine, Busan, South Korea; 5 Cardiovascular Center, Dongguk University Ilsan Hospital, Goyang, South Korea; 6 Division of Cardiology, Department of Internal Medicine, Gangnam Severance Hospital, Yonsei University College of Medicine, Seoul, South Korea; 7 Heart Center of Chonnam National University Hospital, Gwangju, South Korea; 8 Cardiology Division, Department of Internal Medicine, Kangwon National University, School of Medicine, Chuncheon, South Korea; 9 Endocrinology Division, Department of Internal Medicine, Kyung Hee University School of Medicine, Seoul, South Korea; 10 Cardiology Division, Department of Internal Medicine, Keimyung University Dongsan Medical Center, Daegu, South Korea; 11 Cardiology Division, Department of Internal Medicine, School of Medicine, Chungnam National University, Chungnam National University Hospital, Daejeon, South Korea; 12 Department of Clinical Pharmacology and Therapeutics, College of Medicine, Kyung Hee University, 26 Kyungheedae-ro, Dongdaemun-gu, Seoul, 02447 South Korea; 13 Department of Biomedical Science and Technology, Kyung Hee Medical Science Research Institute, Kyung Hee University, Seoul, South Korea

**Keywords:** Clinical genetics, Dyslipidaemias, Risk factors, Dyslipidaemias

## Abstract

Among the 146 patients enrolled in the Korean FH registry, 83 patients who had undergone appropriate LLT escalation and were followed-up for ≥ 6 months were analyzed for pathogenic variants (PVs). The achieved percentage of expected low-density lipoprotein-cholesterol (LDL-C) reduction (primary variable) and achievement rates of LDL-C < 70 mg/dL were assessed. The correlations between the treatment response and the characteristics of PVs, and the weighted 4 SNP-based score were evaluated. The primary variables were significantly lower in the PV-positive patients than in the PV-negative patients (p = 0.007). However, the type of PV did not significantly correlate with the primary variable. The achievement rates of LDL-C < 70 mg/dL was very low, regardless of the PV characteristics. Patients with a higher 4-SNP score showed a lower primary variable (R^2^ = 0.045, p = 0.048). Among evolocumab users, PV-negative patients or those with only defective PVs revealed higher primary variable, whereas patients with at least one null PV showed lower primary variables. The adjusted response of patients with FH to LLT showed significant associations with PV positivity and 4-SNP score. These results may be helpful in managing FH patients with diverse genetic backgrounds.

## Introduction

Many patients with familial hypercholesterolemia (FH) are not diagnosed early enough to be treated properly and this has made it a global health issue^1–3^. Pharmacological treatment for FH has made considerable progress in the past two decades. Currently, pharmacological agents including statins, ezetimibe, and PCSK9 inhibitors are used to treat FH in clinical practice^4,5^. Clinical trials or registry studies conducted till date have reported that individual responses to lipid-lowering therapy (LLT) using statins^6^ and/or PCSK9 inhibitors^7–9^ vary substantially. Individual difference in response to LLT is a crucial clinical issue as it can affect cardiovascular outcomes^10^. However, the reason underlying the variation in response to LLT among individuals is not yet completely understood.

Several clinical^11^ and genetic factors^12^ have been reported to influence the response in general population. Thus, in FH, it is likely that genetic variations affect an individual’s response to LLT. Differences in cardiovascular risk due to genetic characteristics in FH^13,14^ may be partly explained if the presence or type of pathogenic variants (PVs) influenced the patient response. Some studies have reported that the response to statins^15^ and PCSK9 inhibitors^16^ could differ depending on PV types, such as defective PV. However, contradictory studies^8,17^ have warranted the need for further studies to clarify the link between genetic characteristics and responses to statins and PCSK9 inhibitors.

This study aimed to analyze the relationship between genetic characteristics and the response to LLT in patients with FH. The primary variable in our study was the achieved percentage of expected response to LLT. First, the expected low-density lipoprotein-cholesterol (LDL-C) reduction with an LLT regimen was estimated and then the achieved percentage of this expected value was calculated for each subject. The correlation of the 4-SNP score^18^ with the response was also investigated, and the difference in response according to genetic characteristics was evaluated in patients receiving evolocumab.

## Methods

### Study population

Nine university hospitals participated in this study supported by the Korean Society of Lipid and Atherosclerosis^18–21^. All subjects gave written informed consent, and all study protocols were approved by the institutional review board of Severance Hospital, Seoul, Korea. The study protocol conforms to the ethical guidelines of the 1975 Declaration of Helsinki. One hundred and forty-six men and women aged more than 19 years who met the Simon-Broome criteria^22^ for heterozygous FH were enrolled in the study conducted from January 2009 to July 2014. Among them, 83 patients who underwent proper escalation of LLT (described in ‘2.3. Parameters of response to LLT’) and followed up for ≥ 6 months, and for whom pre- and post-treatment LDL-C levels were known were finally analyzed. Among the 63 excluded patients, 31 did not receive appropriate LLT escalation, 18 were not followed-up for 6 months, whereas 14 did not undergo regular measurement of LDL-C.

### Clinical and genetic data collection

Patient history was obtained from every patient, and each patient was subjected to physical examination and laboratory assessment. Patients under LLT at enrollment were asked to skip lipid-lowering agents for 4 weeks unless they had a history of atherosclerotic cardiovascular or cerebrovascular diseases. The patients fasted for 12 h before blood sampling, and the samples were analyzed within 4 h at a local laboratory. DNA sequencing and analysis for putative PVs was performed as described previously^20^ and in Supplementary Information.

According to the detection of non-synonymous variants by the Unified GATK Genotyper (v2.3.6), variants were classified as pathogenic based on the information regarding three FH-related genes on public databases. For variants that had not been previously reported, pathogenicity was confirmed based on one of the following: (1) inevitably deleterious effects of amino acid changes such as frame shift insertions/deletions and copy number deletions or (2) co-segregation of the same variants within the family. Thereafter, all variants listed as pathogenic were validated by Sanger sequencing. All variants were also checked by the MUTALYZER program. Finally, each variant was classified according to the American College of Medical Genetics and Genomics guidelines. Null PVs included point PVs that cause a premature stop codon, missense PVs affecting the fifth cysteine rich repeat in the ligand binding domain of LDLR, small deletions or insertions causing a frame shift, and a premature stop codon or large rearrangements. The remaining in-frame point PVs and in-frame small deletions and insertions consisted of receptor-defective PVs^13^.

### Parameters of response to LLT

When patients were enrolled in the study, LLT was initiated with moderate intensity statins (i.e. rosuvastatin 5‒10 mg, atorvastatin 10‒20 mg, or other statins with a similar efficacy). If the statin was tolerated well, it was up-titrated every 2 months to reach an LDL-C level of 100 mg/dL. Ezetimibe was added if patients were intolerant to the statins or if the target LDL-C level was not achieved with the maximum tolerable dose of statin. Bile acid sequestrants, niacin, or PCSK9 inhibitors were administered to patients who did not show sufficient LDL-C reduction with statin/ezetimibe regimens. However, data only related to treatment responses for maximum statin dose with or without ezetimibe before introduction of other agents were used for the main analysis.

Pre-treatment LDL-C levels were defined as the documented values before drug therapy, whereas post-treatment LDL-C levels were defined as the values obtained by a maximally up-titrated statin/ezetimibe regimen at 6‒12 months after drug treatment. The primary evaluation variable was the achieved percentage of expected LDL-C reduction. The expected LDL-C reduction was calculated using different doses of statins determined from previous studies and reviews (Table 1)^23–29^. Other evaluation variables included the percentage LDL-C reduction and achievement rate of an LDL-C target of < 70 mg/dL.

**Table 1:** Expected LDL-C reduction by different doses of the lipid-lowering regimen.

Lipid-lowering regimen	Expected LDL-C reduction, %
Atorvastatin 10 mg or similar/day	− 40
Atorvastatin 20 mg or similar/day	− 46
Atorvastatin 40 mg or similar/day	− 52
Atorvastatin 80 mg or similar/day	− 56
Atorvastatin 20 mg/ezetimibe 10 mg/day	− 61
Atorvastatin 40 mg/ezetimibe 10 mg/day	− 66
Evolocumab 140 mg/2 weeks	− 54 (additional)

*LDL-C* low-density lipoprotein-cholesterol.

For additional six patients who received evolocumab in addition to the statin/ezetimibe regimens, the achieved percentage of expected LDL-C reduction was analyzed separately. The expected LDL-C reduction after addition of evolocumab to the regimen at a dose of 140 mg/2 weeks for 3 months was assumed to be 54%.

### 4-SNP score

We genotyped the four SNPs associated with cholesterol levels in the FH patients and the general population in East Asia^18,30^, i.e., rs651007, rs599839, rs12654264, and rs2738446 and are located close to *ABO*, *CELSRS*-*PSRC1*-*SORT1*, *HMGCR*, and *LDLR*, respectively. Weighted mean SNP scores of patients were calculated using alleles associated with cholesterol levels and their beta-coefficients, as reported in the East Asian genome-wide association study (Supplementary Table 1. List of SNPs associated with elevated LDL-C levels in East Asians). The correlation between the 4-SNP score and the primary variable was analyzed.

### Statistical analyses

Continuous data are presented as mean ± standard deviation or median (interquartile range), whereas categorical variables are reported as frequencies and percentages. Data related to clinical and laboratory values were compared using the chi-square test. Between-group comparisons were performed using the Mann‒Whitney U-test. Linear regression was used to assess the association between the 4 SNP score and treatment response in the PV-negative group. There were no missing data in this study. All analyses used a significance level of 0.05. SPSS version 25.0 (SPSS Inc., Chicago, IL, USA) was used for the analyses.

## Results

### Clinical characteristics of the study population

The characteristics of the 83 patients enrolled in the study are presented in Table 2. The mean age of the patients was 53 years and 50 (60%) of them were women. Putative PVs were identified in 30 patients (36%) (Supplementary Table S2). The median baseline LDL-C was higher in the carriers of PVs compared to that in the PV-negative patients (246 mg/dL and 206 mg/dL, respectively, p < 0.001). The median baseline LDL-C levels were 256 mg/dL, 248 mg/dL, and 205 mg/dL in patients with null *LDLR* PVs, defective *LDLR* PVs, and *APOB* or *PCSK9* PVs, respectively. However, differences in LDL-C levels between individuals with different PV types were insignificant (Table 3). During the median follow-up period of 10 months, 40 (48%) patients were treated with statin monotherapy, whereas 43 (52%) received statin/ezetimibe combination therapy (Table 2).

**Table 2:** Clinical and laboratory parameters of the study population.

Variables	Value or frequency (total subjects = 83)
Age, years	53 ± 12
Females	50 (60.2)
**Medical history**	
Diabetes mellitus	6 (7.2)
Hypertension	30 (36.1)
Current smoking	2 (2.4)
CAD	27 (32.5)
**Family history**	
Hypercholesterolemia	46 (55.4)
Premature CAD	42 (50.6)
**Physical findings**	
Body mass index, kg/m^2^	24.7 ± 4.5
Tendon xanthoma	17 (20.5)
**Type of FH in clinical diagnosis**	
Definite	18 (21.7)
Possible	65 (78.3)
PV positivity	30 (36.1)
**Lipid profile, mg/dL**	
Total cholesterol	307 (287, 341)
Triglyceride	149 (116, 227)
HDL-C	46 (40, 56)
LDL-C	213 (198, 248)
**Maximal statin-based lipid-lowering regimen**	
Atorvastatin 10 mg or similar	6 (7.2)
Atorvastatin 20 mg or similar	22 (26.5)
Atorvastatin 40 mg or similar	7 (8.4)
Atorvastatin 80 mg or similar	5 (6.0)
Atorvastatin 20 mg/ezetimibe 10 mg or similar	10 (12.0)
Atorvastatin 40 mg/ezetimibe 10 mg or similar	21 (25.3)
Atorvastatin 80 mg/ezetimibe 10 mg or similar	12 (14.5)

Data are presented as n (%), mean ± standard deviation, or median (interquartile range).

Premature CAD is defined as CAD at age < 50 years in a grandparent, aunt, or uncle or at age < 60 years in a parent, sibling, or child.

*CAD* coronary artery disease, *FH* familial hypercholesterolemia, *PV* pathogenic variant, *HDL-C* high-density lipoprotein-cholesterol, *LDL-C* low-density lipoprotein-cholesterol.

**Table 3:** Genetic background and response to LLT with a statin/ezetimibe regimen.

	Total population (n = 83)	PV-negative (n = 53)	PV-positive (n = 30)					p^a^	p^b^	p^c^
			Any PV-positive (n = 30)	*LDLR* mutation (n = 27)			*APOB* or *PCSK9* PV (n = 3)			
				Any *LDLR* PV (n = 27)	Null *LDLR* PV (n = 10)	Defective *LDLR* PV (n = 17)				
Pre-statin/ezetimibe LDL-C, mg/dL	213 (198, 248)	206 (197, 223)	246 (215, 284)	248 (222, 288)	256 (223, 289)	248 (213, 294)	205 (174, –)	< 0.001	0.078	0.63
Post-statin/ezetimibe LDL-C, mg/dL	114 (96, 131)	105 (88, 125)	122 (110, 138)	122 (114, 139)	133 (123, 143)	119 (110, 137)	97 (69, –)	0.012	0.13	0.059
LDL-C reduction, %	51.9 (41.9, 57.3)	50.7 (42.2, 55.6)	50.7 (39.5, 58.4)	50.4 (39.4, 58.8)	49.9 (44.8, 54.4)	51.0 (39.4, 61.2)	59.1 (24.1, –)	0.82	0.56	0.48
LDL-C reduction % of expected value	89.3 (70.1, 109.2)	95.3 (75.3, 118.1)	82.8 (59.1, 91.2)	81.2 (59.9, 91.1)	76.9 (66.6, 85.6)	88.6 (58.0, 94.2)	89.5 (34.5, –)	0.007	0.76	0.15
Achievement of LDL-C < 70 mg/dL (%)	5 (6.0)	4 (7.5)	1 (3.3)	0 (0)	0 (0)	0 (0)	1 (33.3)	0.44	0.003	1.00

Data are presented as median (interquartile range) or number (%).

p^a^: comparison between PV-positive and -negative patients.

p^b^: comparison between *LDLR* PV and *APOB* or *PCSK9* PV carriers.

p^c^: comparison between null *LDLR* PV and defective *LDLR* PV carriers.

*LLT* lipid-lowering therapy, *PV* pathogenic variant, *LDL-C* low-density lipoprotein-cholesterol.

### Genetic variants and response to LLT

In the total study population (n = 83), the median LDL-C decreased from 213 mg/dL to 105 mg/dL (median LDL-C reduction 51.9%). The primary variable, the achieved percentage of expected LDL-C reduction, was 89% and the achievement rate of LDL-C < 70 mg/dL was 6.0%. The distribution of the primary evaluation variable is shown in Supplementary Fig. 1 (Distribution of the achieved percentage of expected LDL-C reduction). The primary variable was significantly lower in the PV-positive patients than in the PV-negative patients (82.8% and 95.3%, respectively, p = 0.007). Although this variable was lower in patients with null *LDLR* PVs than in those with defective *LDLR* PV, the difference was not significant (76.9% and 88.6%, respectively, p = 0.15). The primary variable was similar between carriers of *LDLR* PVs and those with PVs in the other two genes. Only four PV-negative patients and one with *PCSK9* PV achieved LDL-C < 70 mg/dL (Table 3).

### 4-SNP score and response to LLT

**Figure 1:**
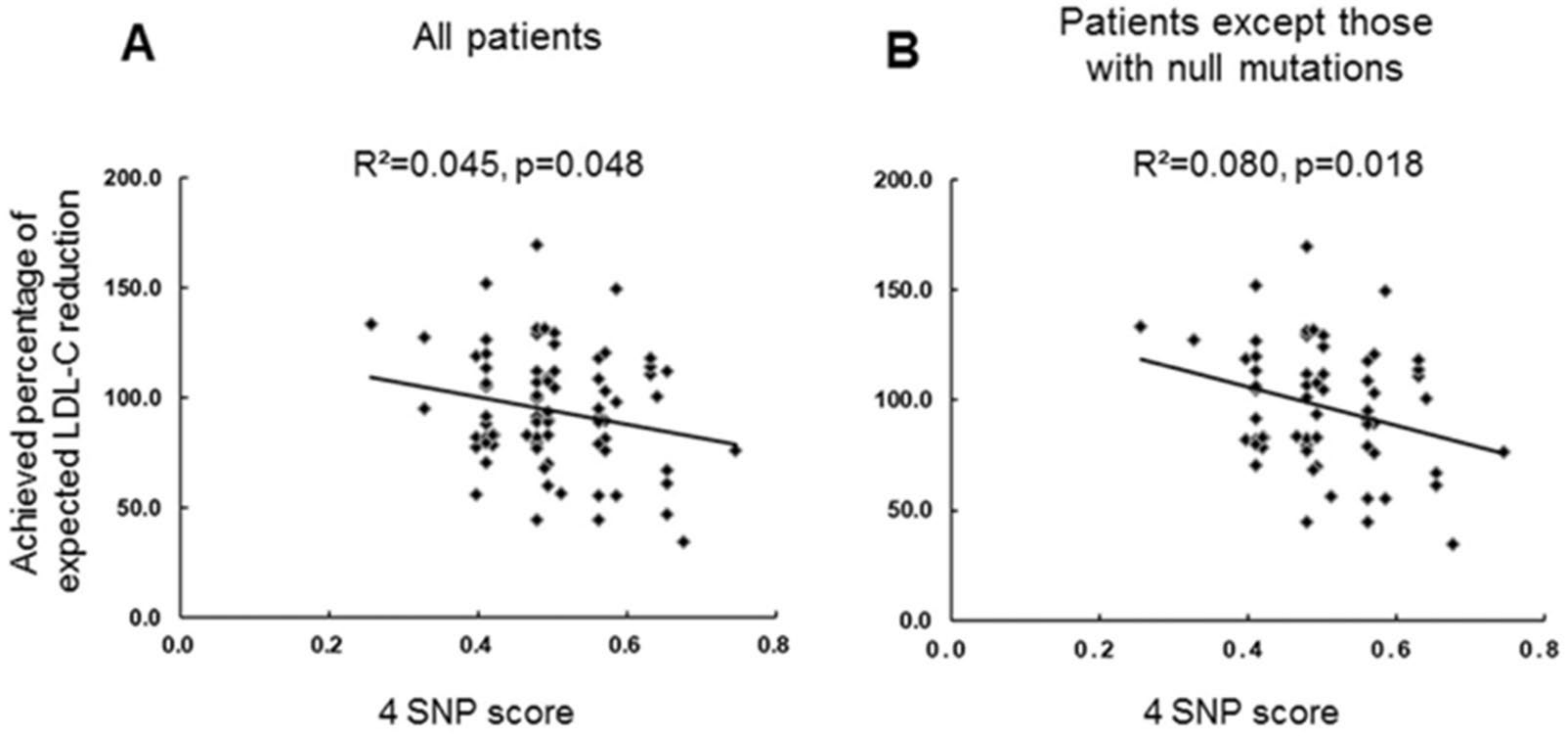
Correlation between the weighted 4-SNP score and the achieved percentage of expected LDL-C reduction in all study patients (**A**) and patients without null PVs (**B**). The image was created using GraphPad Prism version 8.4.3 for Windows (GraphPad Software, San Diego, CA, USA; www.graphpad.com).

The correlation between the weighted mean of the 4-SNP score in the study population and the primary evaluation variable is presented in Fig. 1. Patients with a higher score showed a lower achieved percentage of expected LDL-C reduction (R^2^ = 0.045, p = 0.048) (Fig. 1A). Interestingly, the correlation between the 4-SNP score and the primary variable was stronger in the subgroup of patients without null PVs (R^2^ = 0.080, p = 0.018) (Fig. 1B).

### Genetic variants and response to evolocumab

Six additional patients enrolled and analyzed in the study received evolocumab for ≥ 3 months. One of the patients was PV-negative and the others had PVs in *LDLR*. The primary variable by evolocumab was 153.3% in the PV-negative patients, whereas it was 46.0% in the PV-positive patients. Two of the heterozygous patients had the same null PV in c.682G > T (p.E228X), and the primary evaluation variables were 27.5% and 39.7%, respectively. One heterozygous patient had an *LDLR* copy number variation and a primary variable of 52.2%. The primary variable for the other heterozygous patient with a defective *LDLR* PV was 140.9%. Among the two homozygous patients, one had one null and one defective PV and the other had two defective PVs. Interestingly, the achieved percentages of expected LDL-C reduction were quite different in these two individuals (34.0% and 141.8% in the former and the latter, respectively) (Table 4).

**Table 4:** Genetic background and response to LLT with evolocumab.

	PV-negative (n = 1)	PV-positive (n = 6)						
		Any PV-positive (n = 6)	Heterozygous PV (n = 4)				Homozygous PV (n = 2)	
			c.682G > T (p.E228X) (n = 2)		CNV, exon 8–12 (n = 1)	c.519C > G (C173W) (n = 1)	G558X and c.-136C > T (n = 1)	c.1567G > A (V523M) and c.-136C > T (n = 1)
Pre-evolocuamb LDL-C, mg/dL	157	154 (135, 327)	169	126	138	138	410	299
Post-evolocuamb LDL-C, mg/dL	27	103 (61, 194)	149	99	107	33	329	70
LDL-C reduction, %	82.8	21.9 (17.8, 76.2)	11.8	21.4	22.5	76.1	19.8	76.6
LDL-C reduction, % of expected value	153.3	46.0 (32.4, 141.1)	27.5	39.7	52.2	140.9	34.0	141.8

Data are presented as a number, percentage, or median (interquartile range).

*LLT* lipid-lowering therapy, *PV* pathogenic variant, *LDL-C* low-density lipoprotein-cholesterol, *CNV* copy number variation.

## Discussion

The major findings of this study include the following. First, the adjusted response to LLT, the primary variable, was lower in carriers of PVs than in PV-negative patients. The type of PV did not significantly affect the primary variable. Second, individuals with a higher 4-SNP score showed a lower primary variable. Third, the primary variables for evolocumab-treated patients tended to be lower for PV-positive patients, particularly null PV carriers. We determined the response to LLT according to the adjusted percentage change of LDL-C by regimens titrated in the real world, and analyzed its correlation with the patient’s genotype. Prior studies analyzed response to non-maximal intensity LLT^31^ or unadjusted response to drug doses^32^. Furthermore, some investigators analyzed the response in a binary fashion according to the attainment of target LDL-C levels^15^. We evaluated the relationship between polygenic score and adjusted drug response, which has not been previously reported. In addition, we reported the association between genotype and adjusted response to evolocumab, which has also been rarely discussed in previous studies. However, since the number of our cases on this issue was not large, further studies are needed.

The results of this study confirmed that the response to LLT is poorer in PV-positive patients. PV-positive patients with FH have been known to have higher cardiovascular risk than PV-negative patients^33^. Therefore, genetic information in FH cases could enable risk evaluation, thereby ensuring rigorous management and treatment for individuals with higher risk. Interestingly, 28 patients (33.7%) from our study population received just moderate intensity statins. In additional analysis, the LLT intensities used in the PV-negative group and the PV-positive group were found to be significantly different (p < 0.001) (Supplementary Table S3. LLT intensities used in the PV-negative and -positive patients). In particular, 27 (50.9%) of PV-negative patients used moderate intensity statins, whereas only one (3.3%) of PV-positive patients used moderate intensity statins.

The median percentage change of LDL-C was − 51.9% in our total study population. In clinical trials with heterozygous FH patients, the values were − 49% by pitavastatin 4 mg^34^ and − 46 to − 51% by simvastatin 80 mg^35^. However, in real world data of patients receiving statins with or without ezetimibe, the values varied from − 36^36^ to − 50%^37^. The percentage change of LDL-C of our study was relatively large in our study. However, it is difficult to directly compare these values, as therapeutic regimens were heterogenous in individual studies, and data on the relationship between LLT intensities and responses have not been shown. This is one of the reasons why we used an adjusted parameter of response to LLT.

In the present study, we compared primary variables according to types of PVs. Although the variables were numerically lower in null *LDLR* PV carriers than those in defective *LDLR* PV carriers, the difference was not significant. However, we cannot fully rule out the fact that this might have been significant had the study incorporated a larger number of patients. In a previous study, patients with null PVs had higher LDL-C levels before and after LLT as compared to those with defective PVs^38^ and lower rates of target achievement independent of the statin dose^32^. Furthermore, in a relatively large study conducted on Spanish patients, the presence of defective PVs was found to be an independent predictor of achievement of LDL-C goal^15^. In another study, LDL-C reduction with atorvastatin, 20 mg/day was observed to vary among different classes of *LDLR* PVs (49% and 34% for class V and class II PVs, respectively)^31^. Another study revealed that heterozygous FH patients with defective *LDLR* PVs showed a better response to statin/ezetimibe-based LLT than homozygous FH patients with defective *LDLR* PVs^39^. However, data related to the response to LLT based on types of PVs are inconsistent, as indicated by a previous study^40^, and are not fully understood yet. In a previous study on homozygous FH patients, mean LDL-C reduction by rosuvastatin 20 mg/day was not much different between carriers of defective/negative PVs and those of defective/defective PVs (17.0% and 21.3%, respectively)^17^.

To the best of our knowledge, this study is the first to identify the association between the 4-SNP score and the response to LLT. SNPs that correlated with LDL-C levels in Korean patients with FH in our prior study^18^ were used for the score. A few variants of *SLCO1B1, SLCO1B3,* and *ABCC2* have been reported to be correlated with the response to statins^41^ in Koreans, and this correlation has been linked to pharmacodynamic pathways. In addition, a common loss-of-function variant of *PCSK9* showed an association with greater response to statins in an American study^42^. The Heart Protection Study and a meta-GWAS demonstrated that variants of *SORT1/CELSR2/PSRC1, SLCO1B1, APOE*, and *LPA* are associated with the response to statins^12,43^. The 4-SNP score in this study includes *SORT1, CELSR2,* and *PSRC1*, and the effect of their corresponding variants contributes to the agreement of our findings with those of previous studies.

The present study showed that the presence of a null PV could affect the response to evolocumab regardless of the homozygosity of PVs. Prior data related to the effect of genotype on the response to PCSK9 inhibitors have been variable. An analysis of six studies using alirocumab, a PCSK9 inhibitor, revealed that LDL-C reduction was generally similar across genotypes (54.3% and 60.7% in defective *LDLR*- and negative *LDLR* PV carriers, respectively)^8^. In addition, a recent report on homozygous FH has shown that the response to alirocumab in defective/negative *LDLR* PV carriers was not worse than that in defective/defective *LDLR* PV carriers^44^. On the contrary, in this study, the response to evolocumab was higher than expected in heterozygous FH with defective PVs and in homozygous FH with defective/defective PVs. However, the response in heterozygous and homozygous patients with null PVs was poor. These findings indicate that the influence of null PVs can be greater than that of homozygosity of PVs. A recent study evaluated the long-term effect of evolocumab in homozygous and severe heterozygous FH. Although the relationship between genotype and drug efficacy was not the main focus of this study, reduced efficacy of the agent was observed in patients with two negative *LDLR* PVs^9^.

Our study, however, has potential limitations. First, while the current study used a 4-SNP score, the effect of other SNPs of the same genes or other lipid-related genes cannot be ruled out. Second, because this study was performed using data from Korea, we need to be cautious when generalizing our results. Third, we cannot rule out some difference between patient groups that was not revealed in the current data. For example, median baseline LDL-C levels did not differ according to PV types. However, these were derived in part from limited number of patients with *APOB* or *PCSK9* PVs that usually constitute a small minority of FH patients. However, adjustment of the response to LLT by calculating the achieved percentage of expected LDL-C is the strength of this study.

In conclusion, our findings indicate that adjusted response to LLT has significant associations with PV positivity and the 4-SNP score. The results obtained in this study may ensure effective and individual management of FH patients with diverse genetic backgrounds.

## Supplementary information

Supplementary Information.

## Data Availability

The datasets generated during and/or analyzed during the current study are available from the corresponding author on reasonable request.
