# Supplementary material for: GENetic characteristics and REsponse to lipid-lowering therapy in familial hypercholesterolemia: GENRE-FH study
Source: Sci Rep. 2020 Nov 9;10:19336. doi: 10.1038/s41598-020-75901-0 (PMC7653043; doi:10.1038/s41598-020-75901-0)
Supplement: Supplementary file 1 — Supplementary Information. [file 41598_2020_75901_MOESM1_ESM.pdf]

GENetic characteristics and REsponse to lipid-lowering therapy in Familial Hypercholesterolemia: GENRE-FH study

Hyoeun Kim, Chan Joo Lee, Hayeon Park, Doo-Il Kim, Moo-Yong Rhee, Byoung Kwon Lee, Youngkeun Ahn, Byung-Ryul Cho, Jeong-Taek Woo, Seung-Ho Hur, Jin-Ok Jeong, Ji Hyun Lee, Sang-Hak Lee

SUPPLEMENTARY MATERIAL

Supplementary Methods

Clinical and genetic data collection

Briefly, after genomic DNA was extracted, the DNA sequences of three FH genes (*LDLR*, *APOB*, and *PCSK9*) were obtained by whole-exome sequencing for 65 subjects and by targeted-exome sequencing for the other 18 subjects. For whole-exome sequencing, the Agilent SureSelect Enrichment System (SureSelect All Exon 50 Mb or SureSelect All Exon V4 + UTRs kit; Agilent, Santa Clara, CA, USA) was used. For targeted sequencing, DNA fragments were enriched through solution-based hybridization capture and sequenced on an Illumina HiSeq2500 platform (Illumina, San Diego, CA, USA).

Supplementary Table S1. List of SNPs associated with elevated LDL-C levels in East Asians

rs ID	Chromosome	Nearest gene	Risk allele*	Weight for score calculation (mmol/L)
rs651007	9	*ABO*	A	0.059
rs599839	1	*CELSR2-PSRC1-SORT1*	A	0.135
rs12654264	5	*HMGCR*	T	0.070
rs2738446	19	*LDLR*	G	0.076

*based on dbSNP orientation

Supplementary Table S2. List of pathogenic variants (PVs) found in the study population

Gene	*Base change (AA change)	rs_No	ACMG Classification	No. of patients (n = 30)
LDLR	c.-136C>T	rs879254374	Likely pathogenic	2
	c.268G>A (p.Asp90Asn)	rs749038326	Conflicting interpretations of pathogenicity Likely benign(1); Likely pathogenic(4);Pathogenic(4)	1
	c.285C>A (p.Cys95Ter)	rs139400379	Pathogenic	1
	c.320_332del	rs879254474	Pathogenic	1
	c.418G>A (p.Glu140Lys)	rs748944640	Pathogenic/Likely pathogenic	1
	c.519C>G (p.Cys173Trp)	rs769318035	Pathogenic/Likely pathogenic	2
	c.682G>T (p.Glu228*)	rs121908029	Pathogenic/Likely pathogenic	4
	c.818-2A>G	rs879254687	Pathogenic	1
	c.1216C>T (p.Arg406Trp)	rs121908043	Pathogenic/Likely pathogenic	1
	c.1246C>T (p.Arg416Trp)	rs570942190	Pathogenic/Likely pathogenic	1
	c.1633G>C (p.Gly545Arg)	rs879254965	Likely pathogenic	1
	c.1702C>G (p.Leu568Val)	rs746959386	Pathogenic/Likely pathogenic	1
	c.2054C>T (p.Pro685Leu)	rs28942084	Pathogenic/Likely pathogenic	6

	c.2389G>A (p.Val797Met)	rs750518671	Conflicting interpretations of pathogenicity<br>Likely pathogenic(4);Pathogenic(6);<br>Uncertain significance(3)	1
	c.2500_2502delinsC (p.Asp834fs)	rs879255219	Pathogenic	1
LDLR-CNV	Ex8-12DEL		Pathogenic	1
	Ex1-12DEL		Pathogenic	1
APOB	c.10580G>A (p.Arg3527Gln)	rs5742904	Pathogenic	2
PCSK9	c.94G>A (p.Glu32Lys)	rs564427867	Conflicting interpretations of pathogenicity<br>Likely pathogenic(2);Pathogenic(2);<br>Uncertain significance(2)	1

*Nucleotide location number was assigned according to the *LDLR* (Transcript ID: NM_000527.5), *APOB* (Transcript ID: NM_000384.2) and *PCSK9* (Transcript ID: NM_174936.3) mRNA sequence; AA: amino acid; ACMG: American College of Medical Genetics; PV: pathogenic variant

Supplementary Table S3. LLT intensities used in pathogenic variant (PV)-negative and -positive patients

LLT intensities*	PV-negative (n = 53)	PV-positive (n = 30)	p
Moderate	27 (50.9)	1 (3.3)	<0.001
High	9 (17.0)	3 (10.0)	
Very high	17 (32.1)	26 (86.7)	

LLT: lipid-lowering therapy; PV: pathogenic variant

*Moderate: atorvastatin 10–20 mg or similar; High: atorvastatin 40–80 mg or similar; Very high: atorvastatin 20–80 mg plus ezetimibe 10 mg or similar

Supplementary Figure 1. Distribution of the achieved percentage of expected LDL-C reduction

The image was created using SPSS version 25.0 (SPSS Inc., Chicago, IL, USA)

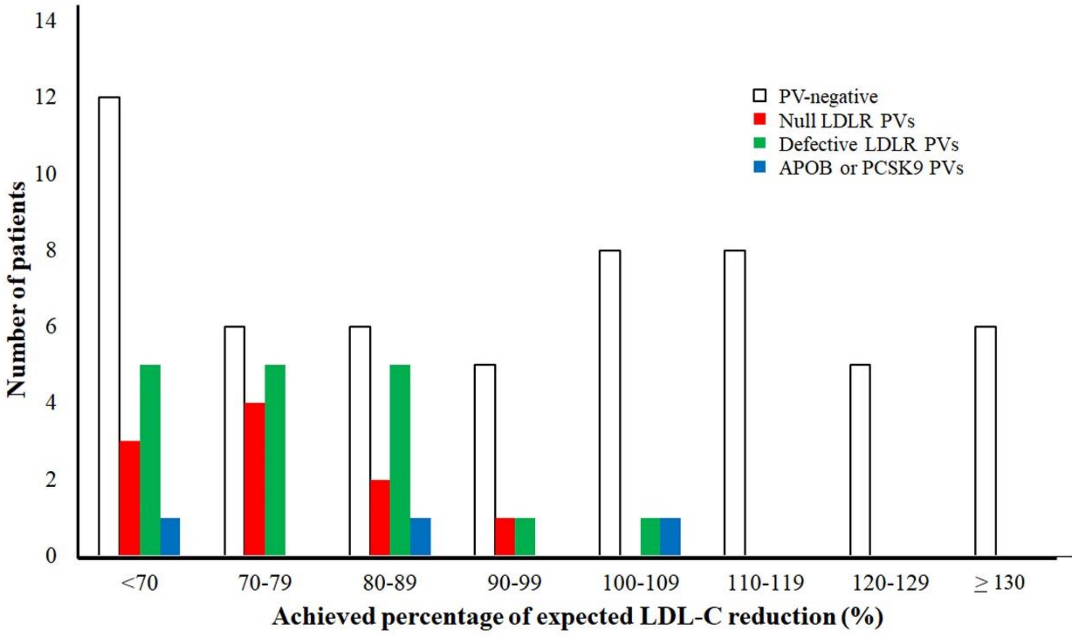

PV: pathogenic variant
